# A Retrospective Study of Ultrasonographic Features of Hepatic Metastases Following Adrenal Cortical Carcinoma Resection

**DOI:** 10.3390/jcm15166442

**Published:** 2026-08-20

**Authors:** Rongchen Wang, Yang Chen

**Affiliations:** Department of Medical Ultrasound, West China Hospital, Sichuan University, Chengdu 610041, China; wangrongchen@wchscu.cn

**Keywords:** adrenocortical carcinoma, liver metastasis, contrast-enhanced ultrasound, conventional ultrasound

## Abstract

**Background/Objectives**: Liver metastasis after surgery for adrenocortical carcinoma (ACC) is a critical factor affecting patient prognosis; however, relevant ultrasound imaging features remain poorly characterized. This retrospective study aims to systematically describe the conventional ultrasound and contrast-enhanced ultrasound (CEUS) features of post-surgical hepatic metastases from ACC and to evaluate the clinical utility of ultrasonography in the diagnosis and follow-up. **Methods**: A total of 10 patients with post-surgical ACC liver metastases via ultrasound-guided liver biopsy between January 2000 and June 2026 at West China Hospital of Sichuan University were retrospectively enrolled. All patients underwent conventional ultrasound (B-mode and color Doppler flow imaging, CDFI) and CEUS. Given the small sample size, only descriptive statistics were performed, and all findings should be interpreted as exploratory. **Results**: All 10 patients were female (age range: 38–56 years), 70% had multiple lesions. On B-mode ultrasound, 80% of lesions appeared hypoechoic, 100% exhibited heterogeneous internal echotexture, 80% had irregular shapes, and 60% displayed well-defined margins. CDFI detected internal or perilesional blood flow signals in 90% of lesions, predominantly perilesional (50%). CEUS demonstrated arterial-phase hyperenhancement in all cases (50% heterogeneous hyperenhancement, 30% peripheral-dominant enhancement, and 20% ring-like nodular hyperenhancement), followed by rapid wash-out during the portal venous or delayed phases, with 100% of lesions showing hypoenhancement at 180 s. **Conclusions**: These exploratory findings suggest that post-surgical ACC liver metastases typically manifest on conventional ultrasound as hypoechoic, heterogeneous solid masses with variable margins and predominant perilesional blood flow. CEUS reveals a characteristic “fast-in, fast-out” malignant enhancement pattern. CEUS may serve as a useful adjunct to conventional imaging within a multimodal surveillance strategy, but larger prospective studies are needed to confirm its diagnostic value.

## 1. Introduction

Adrenocortical carcinoma (ACC) is a rare and highly aggressive malignant endocrine neoplasm arising from the adrenal cortex. Due to its insidious onset, ACC is associated with a low early diagnostic rate and high mortality [1,2].

Epidemiological data indicate that the global incidence of ACC is extremely low, at approximately 1–2 cases per million population [3], accounting for 1% to 2% of all adrenal tumors. It commonly occurs in children aged 1–6 years and adults aged 40–50 years. Most ACC tumors are larger than 5 cm in diameter and tend to invade the renal vein and inferior vena cava. Although ACC accounts for only 0.02% to 0.2% of all cancer-related deaths [4,5], it is characterized by high aggressiveness and functional activity, frequently presenting with virilization in females and adrenal hyperfunction, along with prominent systemic symptoms. The tumor is prone to local invasion and metastasis. Once lymphatic or hematogenous dissemination occurs, the average survival time is generally 2 years. The differentiation between functional and non-functional adrenocortical tumors primarily relies on clinical manifestations, biochemical tests, and hormonal assays. In terms of genetics, the molecular characteristics of ACC were first identified in patients with Birt–Hogg–Dubé syndrome, highlighting the importance of genetic counseling in ACC patients [6]. Furthermore, a genotype–phenotype association has also been observed between Li–Fraumeni syndrome and the development of ACC, underscoring the significance of genetic testing in this patient population [7].

ACC tends to metastasize to the liver, lungs, peritoneum, bones, and lymph nodes at an early stage [2]. For stage I ACC (tumor < 5 cm, confined within the adrenal capsule without invasion), the probability of regional lymph node metastasis is <1%, and the probability of early distant metastasis to the liver, lungs, peritoneum, or bones is <5%. For stage II ACC (tumor ≥ 5 cm, still confined within the adrenal capsule, without vascular invasion), the probability of regional lymph node metastasis is approximately 10%, and the probability of early distant metastasis to the liver, lungs, peritoneum, or bones is 10–20%. For stage III ACC (invasion of surrounding adipose tissue or major vessels, or regional lymph node involvement), the probability of lymph node metastasis is 35–50%, and the probability of occult distant metastasis to the liver, lungs, peritoneum, or bones is 40–60%. In stage IV ACC, defined as the presence of definitively detectable distant metastasis on imaging, one or more metastatic lesions in the liver, lungs, peritoneum, or bones are already present at the time of diagnosis, accounting for 30–40% of initially diagnosed patients. Among stage IV patients, the distribution of metastatic sites is as follows: liver metastasis accounts for 60–70% (the most common distant metastatic site), pulmonary metastasis accounts for 40–50%, peritoneal and abdominal implantation accounts for approximately 30%, and bone metastasis accounts for 20–25%. Furthermore, distant metastasis tends to occur within 2 years after adrenal cortical carcinoma resection. Detailed stage-specific recurrence rates for liver metastasis after complete resection are not consistently reported in the literature; however, the risk is known to increase with higher tumor stage. In summary, the liver is the most common site of ACC metastasis, followed by the lungs and retroperitoneal lymph nodes, while bone metastasis is the least common [8,9].

To date, studies on hepatic metastasis following surgical resection of adrenocortical carcinoma are relatively scarce, with only a few case reports available in the literature. Among the existing studies, the imaging features of liver involvement have been sparsely described, and the radiological evaluation has predominantly relied on computed tomography (CT) or magnetic resonance imaging (MRI) [10]. Ultrasound examination and contrast-enhanced ultrasound (CEUS), as non-invasive, convenient, and repeatable imaging diagnostic modalities, offer advantages including simple operation, absence of ionizing radiation, and low cost, and play an irreplaceable role in the diagnosis, differential diagnosis, and follow-up of hepatic lesions. They can clearly delineate the location, size, morphology, margins, internal echogenicity, blood flow signals, and contrast enhancement patterns of lesions, providing important imaging clues for clinical diagnosis. However, there is currently no consensus regarding the ultrasonographic features of hepatic metastases following adrenal cortical carcinoma resection in clinical practice. Ultrasonographers lack sufficient familiarity with their imaging manifestations, making it difficult to accurately identify and classify these hepatic metastases using conventional ultrasound and CEUS, and thereby failing to provide reliable imaging support for clinical management.

Therefore, this study retrospectively analyzed the ultrasonographic features of hepatic metastases following adrenocortical carcinoma resection in patients diagnosed by ultrasound-guided liver biopsy at West China Hospital of Sichuan University from 2000 to 2026. We recorded and analyzed ultrasound parameters including lesion location, size, morphology, margins, internal echogenicity, blood flow signals, and contrast enhancement patterns, and integrated these findings with clinical data and pathological results to evaluate the value of ultrasound in the diagnosis, classification, and differential diagnosis of postoperative hepatic metastases from adrenocortical carcinoma. This exploratory study aims to improve understanding and diagnostic accuracy of this condition, thereby providing more comprehensive imaging evidence to support clinical diagnosis, treatment planning, and prognostic evaluation.

## 2. Materials and Methods

### 2.1. Patient Data Acquisition

This retrospective study was approved by the Ethics Review Committee of West China Hospital, Sichuan University. A total of 10 patients with pathologically confirmed hepatic metastases following adrenocortical carcinoma resection, diagnosed by ultrasound-guided liver biopsy, were enrolled in this study. Their clinical data were consecutively and retrospectively collected from January 2000 to June 2026. Prior to any imaging examination, all enrolled patients (or the legal guardians of pediatric patients) provided written informed consent and were fully informed of the potential risks and procedural complications associated with the examinations. Comprehensive clinical and pathological characteristics of all patients were obtained from the electronic medical record system. It is important to note that this study’s enrolment required a B-mode-visible lesion amenable to ultrasound-guided biopsy (the smallest lesion in our cohort was 2.6 cm); therefore, these findings do not apply to subcentimetre or sonographically occult metastases.

### 2.2. Image Acquisition

For patients with multiple lesions, only the largest lesion detected on ultrasound was analyzed and reported. Conventional ultrasound (B-mode, color Doppler flow imaging [CDFI]) and CEUS images were both available for evaluation. Ultrasound examinations were performed using the iU22 ultrasound system equipped with a C5-2 (2–5 MHz) convex array probe (Philips Medical Systems, Royal Philips, Amsterdam, The Netherlands), and the Resona 7 ultrasound diagnostic system equipped with a C5-2 convex array probe (frequency: 2–5 MHz) (Mindray Medical International Co., Ltd., Shenzhen, China). All examinations were conducted by sonographers with over 5 years of experience in abdominal ultrasound. Relevant ultrasound parameters (gain, depth, focal zone position) were optimized according to the clinical experience of the sonographers to ensure high-quality imaging of the target lesions. All enrolled patients underwent conventional ultrasound examination, and a systematic assessment of the sonographic findings was performed, including lesion location, margin definition (well-defined vs. ill-defined), morphological regularity (regular vs. irregular), size (maximum longitudinal diameter on the largest cross-sectional plane of the lesion), echogenicity (hypoechoic, hyperechoic, or isoechoic), internal echo uniformity (homogeneous vs. heterogeneous), the presence of a peripheral hypoechoic halo, CDFI signals, and the spatial relationship between the lesion and adjacent anatomical structures. All ultrasound and CEUS images were independently reviewed by two radiologists with >10 years of experience in abdominal imaging, who were blinded to the final pathological diagnosis. In cases of disagreement, a consensus was reached through discussion. Each lesion was assessed for location, size, echogenicity, homogeneity, margin, shape, vascularity on CDFI, and CEUS enhancement patterns (arterial phase: hyperenhancement pattern; portal venous and late phases: washout or hypoenhancement).

Among the enrolled patients, 10 underwent CEUS in addition to conventional ultrasound. During CEUS, a dual-screen mode (simultaneously displaying grayscale ultrasound and CEUS images on the monitor) was employed to enable real-time contrast-enhanced imaging at a low mechanical index (mechanical index set to 0.09), using the Philips iU22 ultrasound system, Mindray Resona 7, and Mindray Resona 9 systems. A suspension of 2.4 mL SonoVue (Bracco, Milan, Italy) was administered via the antecubital vein, followed by a 5 mL saline flush. Timing was initiated immediately after contrast agent injection. The target lesion was continuously observed, and dynamic images were stored in real time for 180 s. The dynamic contrast-enhanced imaging features of the lesion were evaluated and analyzed.

### 2.3. Statistical Analysis

All the statistical analyses were performed using IBM SPSS Statistics Version 27 (IBM Corporation, Armonk, NY, USA). Given the small sample size and the exploratory nature of this study, we employed descriptive statistical methods to summarize the basic characteristics of the study samples, mainly using frequency and percentage to represent the distribution of categorical variables. No inferential statistical tests were performed.

## 3. Results

### 3.1. Clinical Manifestations of Patients

A total of 10 patients were enrolled in this study, all of whom were female. The patients’ age ranged from 38 to 56 years. Seven patients presented with multiple hepatic lesions (7/10, 70%), while three patients presented with a solitary hepatic mass (3/10, 30%); all lesions were located in the liver. In all 10 patients, the lesions were detected incidentally on ultrasound or CT examination without any obvious discomfort, and none of the patients exhibited any relevant clinical symptoms. Routine blood tests, as well as renal and liver function tests, were all within normal reference ranges.

### 3.2. Ultrasonographic Features

All 10 patients underwent conventional ultrasound examination, and all cases received CEUS according to clinical recommendations. All lesions were located in the liver, with the maximum lesion diameter ranging from 2.6 cm to 10.5 cm. All lesions exhibited an ovaloid/round-like morphology. Among them, 6 lesions (6/10, 60%) had well-defined margins, 2 lesions (2/10, 20%) were regular in shape, 4 lesions (4/10, 40%) had ill-defined margins, 8 lesions (8/10, 80%) were irregular in shape, and 2 lesions (2/10, 20%) appeared confluent. In terms of echogenicity, 8 lesions (8/10, 80%) were hypoechoic, while 2 lesions (2/10, 20%) were isoechoic. All 10 lesions (10/10, 100%) demonstrated heterogeneous internal echotexture. On CDFI, 1 lesion (1/10, 10%) showed no discernible blood flow signals, 4 lesions (4/10, 40%) exhibited intralesional and perilesional vascularity, and 5 lesions (5/10, 50%) showed perilesional blood flow signals only.

**Figure 1:**
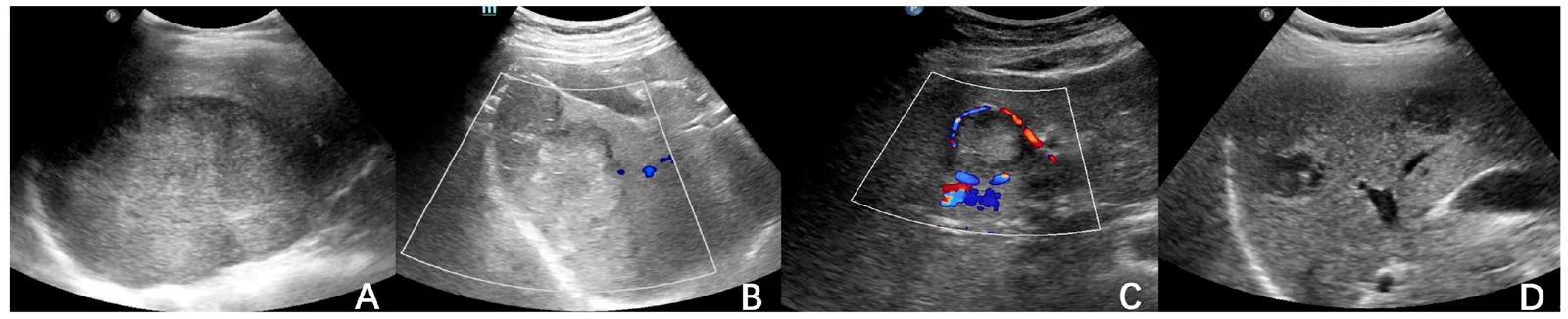
Representative conventional ultrasound images of hepatic metastases following adrenocortical carcinoma resection. (**A**) Solitary hepatic lesion with well-defined margins and regular morphology. (**B**) Hepatic lesion with well-defined margins, irregular contour, and absence of detectable vascularity on CDFI. (**C**) Hepatic lesion demonstrating peripheral rim-like flow signals on color Doppler imaging. (**D**) Multiple hepatic lesions.

**Figure 2:**
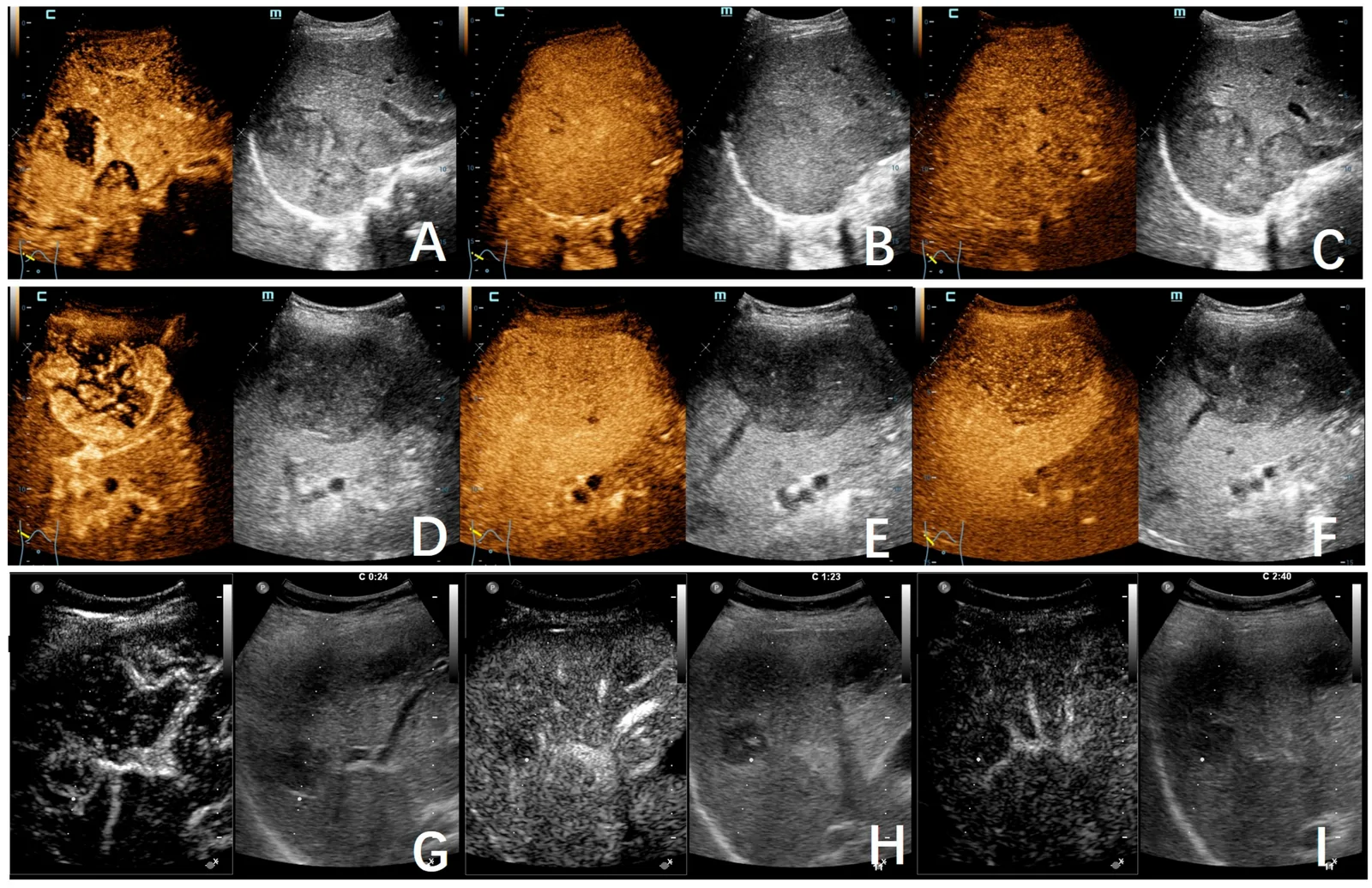
CEUS images of hepatic metastases from adrenocortical carcinoma, demonstrating three representative enhancement patterns. (**A**–**C**) Pattern 1: heterogeneous hyperenhancement in the arterial phase (**A**), early washout in the portal venous phase (**B**), and hypoenhancement in the late parenchymal phase (**C**). (**D**–**F**) Pattern 2: predominantly peripheral hyperenhancement in the arterial phase with early washout seen in the late arterial phase (**D**), followed by hypoenhancement in the portal venous phase (**E**) and late parenchymal phase (**F**). (**G**–**I**) Pattern 3: rim-like nodular hyperenhancement in the arterial phase (**G**), with hypoenhancement in the portal venous phase (**H**) and late parenchymal phase (**I**).

In all 10 patients, initial conventional ultrasound raised suspicion of malignant lesions. Consequently, CEUS was performed in addition to standard ultrasound evaluation per clinical recommendation. CEUS was successfully completed in all 10 patients with satisfactory image quality. Following intravenous administration of the ultrasound contrast agent SonoVue, contrast-enhanced imaging revealed the following arterial-phase enhancement patterns: heterogeneous hyperenhancement in 5 cases (5/10, 50%), predominantly peripheral hyperenhancement in 3 cases (3/10, 30%), and rim-like nodular hyperenhancement in 2 cases (2/10, 20%). Early washout began in the portal venous phase in 6 cases (6/10, 60%), and hypoenhancement was observed in the portal venous phase in 4 cases (4/10, 40%). All 10 cases (10/10, 100%) demonstrated hypoenhancement at 180 s. Based on the CEUS findings in conjunction with clinical history, all 10 cases were considered to be consistent with metastatic tumors. The conventional ultrasound and contrast-enhanced ultrasound findings of all enrolled patients are shown in Table 1. Representative conventional ultrasound images are presented in Figure 1, and three different patterns of contrast-enhanced ultrasound findings are presented in Figure 2. Supplementary Tables S1 and S2 provide detailed information on the distribution of examination dates and times for the ultrasound systems used.

### 3.3. Histopathological Features and Follow-Up

All 10 patients underwent ultrasound-guided tumor biopsy. Pathological examination of the biopsy specimens revealed atypical cells in all cases, with a recommendation for immunohistochemical staining to aid in diagnosis. Immunohistochemistry demonstrated the following staining profile: α-Inhibin (+), Ki67 (MIB-1) (+, 10–15%), p53 (+), MART-1 (+), SF-1 (+), Melan-A (+), and HepPar1/Arg-1 (−). Based on the integration of clinical history, histological morphology, and immunohistochemical findings, all 10 cases were consistent with hepatic metastases following adrenocortical carcinoma resection.

## 4. Discussion

ACC is a rare but highly aggressive malignant endocrine neoplasm, and postoperative hepatic metastasis represents a critical determinant of long-term patient survival [1,2]. Although ACC accounts for an extremely low proportion of all cancer-related deaths, its high recurrence rate and propensity for early distant metastasis render it a formidable challenge in clinical management. In this retrospective study, we systematically analyzed the ultrasonographic features of 10 pathologically confirmed cases of postoperative hepatic metastasis from ACC, providing a detailed characterization of the typical conventional ultrasound (B-mode and CDFI) and CEUS findings in this patient population. Our exploratory findings suggest that ultrasound, particularly CEUS, holds clinical value in the detection and characterization of ACC hepatic metastases, and can provide useful imaging evidence to facilitate early diagnosis and guide subsequent therapeutic decision-making. However, given the small sample size and retrospective design, these observations should be considered hypothesis-generating.

All 10 patients enrolled in this study were female, with ages ranging from 38 to 56 years, a distribution consistent with the epidemiological features of ACC, which predominantly affects middle-aged adults in their 40s to 50s [4,5]. In all cases, hepatic lesions were incidentally detected on imaging during routine follow-up, without any gastrointestinal or liver function-related clinical symptoms, and hematological parameters were within normal limits. These findings indicate that postoperative hepatic metastases from ACC are notably insidious, and early metastatic foci may be insufficient to cause hepatic decompensation or specific symptoms. Therefore, regular imaging-based surveillance following surgery is of paramount importance for ACC patients [8,10].

In terms of conventional ultrasound findings, the cases in this group exhibited certain common characteristics. The majority of lesions (80%) were hypoechoic, and all lesions (100%) demonstrated heterogeneous internal echoes, which is consistent with the non-homogeneous growth pattern typical of most malignant hepatic tumors. Notably, the lesions showed considerable variability in morphology and margin delineation: although most lesions were irregular in shape (80%) and ill-defined (40%), a considerable proportion also had well-defined margins (60%) or even regular morphology (20%). This diversity may be attributable to differences in tumor growth patterns, the formation of fibrous capsules, or compression effects from the surrounding hepatic parenchyma. CDFI revealed that only 10% of the lesions lacked detectable blood flow signals, while 90% demonstrated intralesional or perilesional vascularity, with perilesional predominance (50%). This “peripherally hypervascular” pattern may represent a common feature of metastatic tumors, as the blood supply to metastases often relies on passive perfusion from host liver vasculature, with the tumor periphery being the site of most active invasion and angiogenesis. Although these conventional ultrasound features provide some suggestive clues, they lack high specificity and are insufficient to reliably differentiate ACC hepatic metastases from hepatocellular carcinoma (HCC) or other hypervascular metastases (e.g., neuroendocrine tumor metastases) based solely on B-mode ultrasound and CDFI.

The application of CEUS significantly improved diagnostic confidence in our cohort. In this study, all 10 patients exhibited a typical “malignant” enhancement pattern on CEUS, characterized by rapid, heterogeneous hyperenhancement or rim-like/nodular hyperenhancement in the arterial phase, followed by rapid washout in the portal venous or late phase. Specifically, 50% of cases showed heterogeneous arterial-phase hyperenhancement with early portal venous washout, 30% showed predominantly peripheral enhancement, and 20% showed rim-like nodular hyperenhancement. Notably, all cases (100%) demonstrated hypoenhancement at 180 s. This “fast-in, fast-out” enhancement pattern is a hallmark of malignancy, with the underlying pathophysiological basis being the structurally disordered neovasculature and increased vascular permeability within the tumor, resulting in rapid contrast agent entry and subsequent swift washout [11]. Unlike the classic “fast-in, slow-out” or “fast-in, iso-out” pattern observed in HCC, ACC hepatic metastases exhibit more rapid and complete washout, a distinction of considerable value for differential diagnosis. Furthermore, the heterogeneous arterial-phase enhancement reflects the frequent presence of internal heterogeneity, including necrosis, hemorrhage, or fibrosis, within ACC metastatic lesions, which corresponds to the histopathological features of tumor cells arranged in nested patterns with abundant stromal vasculature and frequent central necrosis. The heterogeneous echotexture and heterogeneous arterial enhancement observed on CEUS correspond histopathologically to the frequent presence of necrosis, hemorrhage, and fibrovascular stroma within ACC metastases, as confirmed by the biopsy findings in our cohort.

It is important to note that the “fast-in, fast-out” pattern is not pathognomonic for ACC metastases; it is also observed in other hypervascular liver metastases (e.g., neuroendocrine tumors, renal cell carcinoma) and some hepatocellular carcinomas. Therefore, CEUS findings should always be interpreted in conjunction with clinical history and other imaging modalities.

From the perspective of tumor staging and metastatic risk, the occurrence of postoperative hepatic metastases in our cohort was closely correlated with the initial stage of the primary tumor. In the present study, the majority of patients presented with multiple lesions (70%), with the maximum lesion diameter reaching up to 10.5 cm, suggesting that the included patients were predominantly at high-risk stages or already in an advanced disease phase. Furthermore, immunohistochemical analysis in all cases demonstrated positivity for Ki-67 (MIB-1) (10–15%), p53, SF-1, and Melan-A, with negativity for HepPar-1 and Arg-1. This panel of markers not only confirmed the adrenocortical origin of the metastases but also effectively excluded hepatocellular carcinoma, further underscoring the necessity of a multimodal diagnostic approach (integrating imaging, pathology, and immunohistochemistry) in the workup of challenging cases.

However, this study has several important limitations. First, as a retrospective study, the sample size was limited to only 10 cases, which restricts the statistical power and the feasibility of subgroup analyses, making it difficult to evaluate the association between specific ultrasound features and patient prognosis. Therefore, the percentages reported herein are descriptive only and should not be overinterpreted as precise estimates. These observations are hypothesis-generating and require validation in larger cohorts. Second, the enrolment was restricted to patients with B-mode-visible lesions amenable to ultrasound-guided biopsy (smallest diameter 2.6 cm); consequently, our findings cannot be extrapolated to subcentimetre or sonographically occult metastases. Third, the retrospective, single-center design and the extended recruitment period (2000–2026) introduce potential selection bias and heterogeneity in imaging protocols, equipment, and operator expertise over time. The temporal distribution of the ultrasound systems used is shown in Supplementary Tables S1 and S2; however, the small numbers preclude any formal analysis of system-related differences, and this technological heterogeneity should be considered a limitation when comparing findings across the study period. Fourth, CEUS evaluation relies on the operator’s experience and subjective interpretation, and lacks fully objective quantitative parameters (such as time–intensity curve analysis), which may introduce a certain degree of observer bias. Interobserver variability was not formally assessed, which is a limitation given the qualitative nature of CEUS interpretation. Additionally, the CEUS observation window was limited to 180 s, whereas standard liver CEUS protocols often extend the late phase to 4–6 min. Therefore, our finding of 100% hypoenhancement at 180 s may not fully capture the complete late-phase behavior, and longer observation periods could potentially reveal different enhancement patterns. Future multicenter, large-sample prospective studies, incorporating elastography and artificial intelligence-assisted analysis, are warranted to further refine the ultrasound diagnostic criteria for postoperative hepatic metastases from ACC.

In summary, conventional ultrasound of hepatic metastases following adrenocortical carcinoma resection typically demonstrates hypoechoic, heterogeneous solid masses with variable margin definition, and CDFI frequently reveals perilesional blood flow signals. CEUS characteristically shows rapid heterogeneous hyperenhancement in the arterial phase, followed by rapid washout in the portal venous and late phases. When these features are combined with a known history of ACC surgery, they are highly suggestive of postoperative hepatic metastases from ACC. Ultrasound, particularly CEUS, as a non-invasive, convenient, and radiation-free imaging modality, may be considered a useful adjunct within a multimodal surveillance strategy for ACC patients. CEUS is not intended to replace CT or MRI but rather to provide additional functional information that may aid in lesion characterization. It plays a valuable clinical role in the early detection of hepatic metastases, guidance of liver biopsy, and assessment of treatment response. Clinicians should enhance their awareness of these imaging features to optimize the comprehensive management strategies for patients following ACC resection.

## 5. Conclusions

In conclusion, the incidence of hepatic metastases following adrenocortical carcinoma resection is extremely low, which may easily lead to clinical misdiagnosis or delayed treatment, particularly when clinical and imaging presentations are atypical. These exploratory findings suggest that ultrasound and CEUS can serve as reliable adjunctive tools for the diagnosis of postoperative hepatic metastases from ACC. On conventional ultrasound, when a solid liver mass with well-defined margins, irregular shape, and heterogeneous hypoechogenicity is detected in a middle-aged female with a history of ACC, ACC hepatic metastasis should be strongly suspected. CEUS demonstrates rapid heterogeneous hyperenhancement or rim-like/nodular hyperenhancement in the arterial phase, followed by rapid washout in the portal venous or late phase. This “fast-in, fast-out” enhancement pattern, a hallmark of malignancy, may increase diagnostic confidence for ACC liver metastases, particularly in atypical cases. Larger prospective studies are needed to confirm these findings and establish definitive diagnostic criteria.

## Figures and Tables

**Table 1:** Conventional ultrasound and CEUS findings grouped by CEUS arterial enhancement pattern.

Arterial-Phase Enhancement Pattern	B-Mode			Color Doppler Flow Imaging	Portal Venous Phase	Late Phase (180 s)
	Echogenicity	Margin	Shape			
Heterogeneous hyperenhancement (n = 5)	Hypoechoic (4), isoechoic (1); Heterogeneous (5)	Clear (4) Ill-defined (1)	Regular (1) Irregular (4)	Peripheral (3) Internal + Peripheral (2)	Early washout (5)	Hypoenhancement (5)
Predominantly peripheral hyperenhancement (n = 3)	Hypoechoic (2), isoechoic (1) Heterogeneous (3)	Clear (1) Ill-defined (2)	Irregular (3)	Internal + Peripheral (2) Peripheral (1)	Early washout (1) Hypoenhancement (2)	Hypoenhancement (3)
Rim-like nodular hyperenhancement (n = 2)	Hypoechoic (2); Homogeneous (1), heterogeneous (1)	Clear (1) Ill-defined (1)	Regular (1) Irregular (1)	None (1) Peripheral (1)	Hypoenhancement (2)	Hypoenhancement (2)

## Data Availability

The original contributions presented in this study are included in the article. Further inquiries can be directed to the corresponding author.

## Supplementary Materials

The following supporting information can be downloaded at https://www.mdpi.com/article/10.3390/jcm15166442/s1. Table S1: Examination date, ultrasound system, and probe used for each case. Table S2: Temporal distribution of ultrasound examinations by system.

## Abbreviations

ACC	Adrenocortical carcinoma
CEUS	Contrast-enhanced ultrasound
CDFI	Color Doppler flow imaging
CT	Computed tomography
MRI	Magnetic resonance imaging
HCC	Hepatocellular carcinoma

## References

[1] Verlinde L., Kinet S., Klaas V.D.H., Brusselaers N., Van Slycke S. (2025). Adrenal cortical carcinoma—A case series and literature review of aggressive adrenal incidentalomas. Acta Chir. Belg..

[2] Taieb A., Yanes A., Fredj R.B., Barkache M., Zarrouk O., Saafi W., Halloul I., El Fekih H., Lajmi Z., Ben Romdhane Y. (2026). Cerebral Metastasis in a Fatal Adrenocortical Carcinoma: A Rare Presentation of an Aggressive Tumor. Diagnostics.

[3] Ettaieb M., Kerkhofs T., van Engeland M., Haak H. (2020). Past, Present and Future of Epigenetics in Adrenocortical Carcinoma. Cancers.

[4] Pronio A., Piroli S., Ciamberlano B., De Luca A., Marullo A., Barretta A., Mazzesi G., Rossi M., Montesani C. (2015). Adrenocortical carcinoma with inferior vena cava, left renal vein and right atrium tumor thrombus extension. Int. J. Surg. Case Rep..

[5] Ayati M., Shahbazi J., Tehranchi A., Ayati E., Rezaei Y. (2015). Adrenocortical Carcinoma with Renal Vein Thrombus Extended to Inferior Vena Cava: A Case Report. Int. Surg..

[6] Kim D., Murvelashvili N., Hamidi O., Jia L. (2023). Adrenal Cortical Carcinoma and Additional Rare Pathologic Findings in Multi-Organs in a Birt-Hogg-Dubé Syndrome Patient: With an Emphasis on the Molecular Characteristics of Adrenal Cortical Carcinoma. Int. J. Surg. Pathol..

[7] Penkert J., Strüwe F.J., Dutzmann C.M., Doergeloh B.B., Montellier E., Freycon C., Keymling M., Schlemmer H.P., Sänger B., Hoffmann B. (2022). Genotype-phenotype associations within the Li-Fraumeni spectrum: A report from the German Registry. J. Hematol. Oncol..

[8] Libé R. (2015). Adrenocortical carcinoma (ACC): Diagnosis, prognosis, and treatment. Front. Cell Dev. Biol..

[9] David S.O., Krieg S., Esposito I., Schott M., Giesel F.L., Roderburg C., Loosen S.H., Luedde T., Knoefel W.T., Krieg A. (2023). A Revised Version of the TNM Classification Leads to Optimized Predictive Performance in Patients with Adrenocortical Carcinoma. Horm. Metab. Res..

[10] Fassnacht M., Dekkers O.M., Else T., Baudin E., Berruti A., de Krijger R., Haak H.R., Mihai R., Assie G., Terzolo M. (2018). European Society of Endocrinology Clinical Practice Guidelines on the management of adrenocortical carcinoma in adults, in collaboration with the European Network for the Study of Adrenal Tumors. Eur. J. Endocrinol..

[11] Weis S.M., Cheresh D.A. (2005). Pathophysiological consequences of VEGF-induced vascular permeability. Nature.

