# Supplementary material for: A Retrospective Study of Ultrasonographic Features of Hepatic Metastases Following Adrenal Cortical Carcinoma Resection
Source: J Clin Med. 2026 Aug 20;15(16):6442. doi: 10.3390/jcm15166442 (PMC13513468; doi:10.3390/jcm15166442)
Supplement: Supplementary file 1 [file jcm-15-06442-s001.zip › jcm-4449633-supplementary.pdf]

Supplementary Table S1. Examination date, ultrasound system, and probe used for each case.

Case No.	Examination Year	Ultrasound System	Probe
1	2017	Philips iU22	C5-2 convex
2	2016	Mindray Resona 7	C5-2 convex
3	2018	Philips iU22	C5-2 convex
4	2015	Mindray Resona 7	C5-2 convex
5	2020	Philips iU22	C5-2 convex
6	2014	Mindray Resona 7	C5-2 convex
7	2022	Mindray Resona 9	C5-2 convex
8	2019	Philips iU22	C5-2 convex
9	2024	Mindray Resona 9	C5-2 convex
10	2008	Philips iU22	C5-2 convex

Supplementary Table S2. Temporal distribution of ultrasound examinations by system.

Ultrasound System	2008–2012	2013–2017	2018–2022	2023–2026	Total
Philips iU22	1	1	3	0	5
Mindray Resona 7	0	3	0	0	3
Mindray Resona 9	0	0	1	1	2
Total	1	4	4	1	10
